# Identification of Moesin (MSN) as a Potential Therapeutic Target for Colorectal Cancer via the β-Catenin-RUNX2 Axis

**DOI:** 10.3390/ijms241310951

**Published:** 2023-06-30

**Authors:** Chien-Yu Huang, Po-Li Wei, Uyanga Batzorig, Precious Takondwa Makondi, Cheng-Chin Lee, Yu-Jia Chang

**Affiliations:** 1 School of Medicine, National Tsing Hua University, Hsinchu 30013, Taiwan; cyhuang@life.nthu.edu.tw; 2 Institute of Molecular and Cellular Biology, National Tsing Hua University, Hsinchu 30013, Taiwan; 3 Department of Pathology, Wan Fang Hospital, Taipei Medical University, Taipei 11696, Taiwan; 4 Department of Surgery, School of Medicine, College of Medicine, Taipei Medical University, Taipei 11031, Taiwan; poliwei@tmu.edu.tw; 5 Division of Colorectal Surgery, Department of Surgery, Taipei Medical University Hospital, Taipei Medical University, Taipei 11031, Taiwan; 6 Cancer Research Center and Translational Laboratory, Department of Medical Research, Taipei Medical University Hospital, Taipei Medical University, Taipei 11031, Taiwan; 7 Graduate Institute of Cancer Biology and Drug Discovery, Taipei Medical University, Taipei 11031, Taiwan; 8 Department of Dermatology, University of California, San Diego, CA 92093, USA; buyangaaa@yahoo.com; 9 Kamuzu Central Hospital, National Cancer Center, Lilongwe P.O. Box 149, Malawi; 10 Graduate Institute of Medical Sciences, College of Medicine, Taipei Medical University, Taipei 11031, Taiwan; kerwinpipi@gmail.com; 11 Graduate Institute of Clinical Medicine, College of Medicine, Taipei Medical University, Taipei 11031, Taiwan; 12 Cell Physiology and Molecular Image Research Center, Wan Fang Hospital, Taipei Medical University, Taipei 11696, Taiwan

**Keywords:** moesin, colorectal cancer, *RUNX2*, β-*catenin*, GSK3β, proliferation, migration, invasion

## Abstract

CRC is the second leading cause of cancer-related death. The complex mechanisms of metastatic CRC limit available therapeutic choice. Thus, identifying new CRC therapeutic targets is essential. Moesin (MSN), a member of the ezrin–radixin–moesin family, connects the cell membrane to the actin-based cytoskeleton and regulates cell morphology. We investigated the role of MSN in the progression of CRC. GENT2 and oncomine were used to study MSN expression and CRC patient outcomes. MSN-specific shRNAs or MSN-overexpressed plasmid were used to establish MSN-KD and MSN overexpressed cell lines, respectively. SRB, migration, wound healing, and flow cytometry were used to test cell survival and migration. Propidium iodide and annexin V stain were used to analyze the cell cycle and apoptosis. MSN expression was found to be higher in CRC tissues than in normal tissues. Higher MSN expression is associated with poor overall survival, disease-free survival, and relapse-free survival rates in CRC patients. MSN silencing inhibits cell proliferation, adhesion, migration, and invasion in vitro, whereas MSN overexpression accelerates cell proliferation, adhesion, migration, and invasion. RNA sequencing was used to investigate differentially expressed genes, and RUNX2 was discovered as a possible downstream target for MSN. In CRC patients, RUNX2 expression was significantly correlated with MSN expression. We also found that MSN silencing decreased cytoplasmic and nuclear β-catenin levels. Additionally, pharmacological inhibition of β-catenin in MSN-overexpressed cells led to a reduction of RUNX2, and activating β-catenin signaling by inhibiting GSK3β rescued the RUNX2 downregulation in MSN-KD cells. This confirms that MSN regulates RUNX2 expression via activation of β-catenin signaling. Finally, our result further determined that RUNX2 silencing reduced the ability of MSN overexpression cells to proliferate and migrate. MSN accelerated CRC progression via the β-catenin-RUNX2 axis. As a result, MSN holds the potential to become a new target for CRC treatment.

## 1. Introduction

Colorectal cancer (CRC) is the world’s second leading cause of cancer-related death, with CRC cases expected to rise by 60% to more than 2.2 million by 2030 [1,2,3,4]. Patients with CRC have a 5-year survival rate of around 65% in some high-income countries, but less than 50% in low-income countries [5,6,7]. Molecular markers identified in the last decade have played an essential role in the early diagnosis and treatment of CRC [8]. APC, β-catenin (CTNNB1), KRAS, BRAF, SMAD4, transforming-growth factor-beta receptor 2, TP53, phosphatidylinositol-4,5-bisphosphate 3-kinase catalytic subunit-alpha, AT-rich interactive domain 1A, sex-determining region Y box 9, family with sequence similarity 123B (also known as AMER1), and ERBB2 have been identified as the most common alterations involved in CRC tumorigenesis [9]. Surgery, chemotherapy, targeted therapy, and radiotherapy are all treatment options for CRC [10,11]. Despite the use of various treatment modalities for CRC management, the outcome is still unsatisfactory, particularly for patients with advanced CRC. As a result, identifying potential markers involved in CRC pathogenesis may allow for the development of treatments that target the specific patients.

Moesin (MSN) belongs to the ezrin–radixin–moesin (ERM) family consisting of talin, ezrin, radixin, protein 4.1., and merlin [12,13,14]. MSN links the cell membrane and actin-based cytoskeleton and is involved in controlling cell morphology [12]. MSN is expressed in the basal layers of the squamous epithelium and glandular ducts and by lymphocytes, and it is highly expressed in the endothelium of blood vessels. Moreover, MSN is involved in invasion, migration, and drug resistance in different types of cancer [15,16,17,18,19,20,21]. The expression of MSN is correlated with the histological grade, development, and recurrence of breast cancer [22,23]. A high MSN expression level in patients with estrogen receptor-positive breast cancer who were treated with tamoxifen and anthracycline alone or in combination with paclitaxel chemotherapy was associated with a low survival rate [24,25]. MSN mediates pancreatic cancer progression by increasing the matrix metalloproteinase (MMP)-7 level, inducing the release of tumor necrosis factor-α and interleukin (IL)-6 and attenuating the IL-10 level [8,26]. Sikorska et al. reported that podoplanin plays a role in the epithelial–mesenchymal transition (EMT) by regulating the expression of ezrin, radixin, and MSN in association with MMPs in thyroid carcinoma cells [27]. Furthermore, the high expression level of MSN was correlated with the aggressive orthotopic growth of glioblastoma in nude mice [28]. A proteomic CRC study demonstrated that MSN was not expressed in the normal colorectal epithelium [29]. Another histological study reported that MSN is highly expressed in the stroma of CRC tissues and is correlated with early Duke’s stages [30]. However, the role of MSN remains unclear.

Runt-related transcription factor (RUNX) family proteins are categorized into three types: RUNX1, RUNX2, and RUNX3 [31]. Among them, RUNX2 is a well-known protein involved in the regulation of cell proliferation and differentiation in the bone [32]. RUNX2 promotes the EMT process and sphere formation in CRC, and its high expression is correlated with metastatic CRC and poor survival [33,34]. RUNX2 is highly correlated with CIMP+/KRAS2 alterations in colon tumors [35]. Another study indicated that RUNX2 may serve as a biomarker for the prediction of CRC recurrence and metastasis [36]. Furthermore, the RUNX2–PVT1–miR-455 regulatory axis plays a critical role in CRC tumorigenesis [37]. Loss of RUNX2 expression caused resistance to mitogen-activated protein kinase/extracellular signal-regulated kinase (MEK) inhibitors through receptor tyrosine kinases (RTKs) in CRC tumors with KRAS alterations [38]. Furthermore, high MSN expression activated the Wnt/β-catenin pathway and resulted in aggressive orthotopic glioblastoma development in mice [28].

This study investigated the role of MSN in CRC proliferation and metastasis. Our findings revealed that the MSN level was highly correlated with the overall survival, disease-free survival, and relapse-free survival of CRC patients. Silencing of MSN reduced the growth, migration, and invasion of CRC cells. Furthermore, we determined that MSN may regulate RUNX2 through the β-catenin/WNT signaling pathway. Our findings indicate that MSN may be a novel therapeutic target for CRC.

## 2. Results

### 2.1. MSN Was Found to Be Upregulated in Cancer Tissues and Predicted a Poor Prognosis in CRC Patients

**Figure 1:**
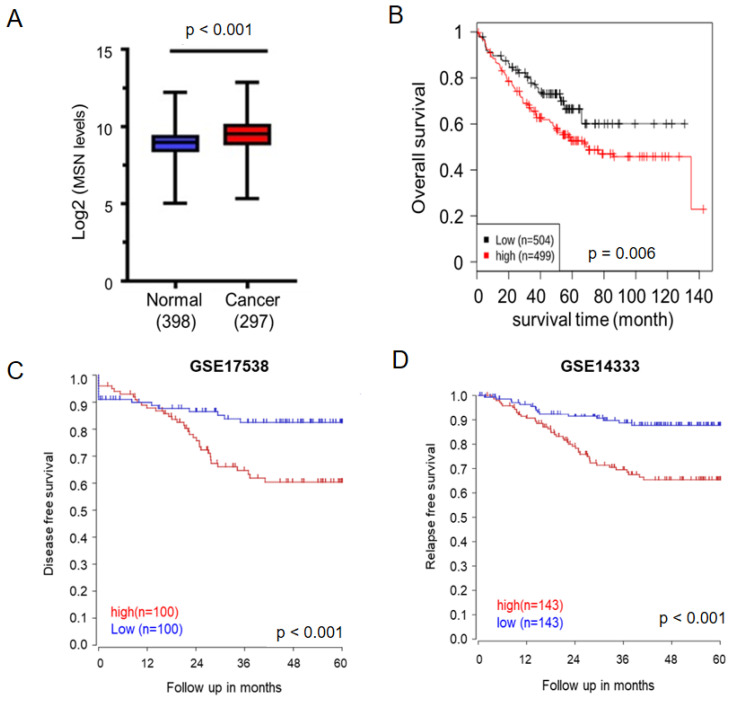
Elevated MSN expression in tumors predicts unfavorable prognosis in patients with colorectal cancer (CRC). (**A**) Expression of MSN in patients with CRC (*n* = 297) and healthy individuals (*n* = 398) was analyzed using the GENT2 database. A significant difference was observed between the normal and cancer tissues (*p* < 0.0001). (**B**) The prognostic value of MSN was analyzed using the GENT2 database. Patients were divided into two groups according to the median expression value. A level below the median value was defined as low expression, and a level above the median value was defined as high expression. High expression of MSN was significantly associated with poor overall survival in patients with CRC (*p* = 0.006). (**C**) Disease-free survival was analyzed from GSE17538. High expression of MSN was significantly associated with disease-free survival in patients with CRC (*p* < 0.001). (**D**) Relapse-free survival was analyzed from GSE14333. High MSN expression was significantly associated with disease-free survival in patients with CRC (*p* < 0.001).

Previous research has found that MSN is upregulated in CRC tissues. We used GENT2 and the R2 Platform to examine the relationship between MSN expression and CRC prognosis to see if MSN regulates CRC progression. MSN expression levels were significantly higher in CRC tissues (*n* = 297) than in normal tissues (*n* = 398; Figure 1A), similar to previous studies. Furthermore, in CRC patients, lower MSN expression was associated with better overall survival, disease-free survival, and relapse-free survival (Figure 1B–D).

### 2.2. CRC Cell Growth Was Mediated by MSN Expression

**Figure 2:**
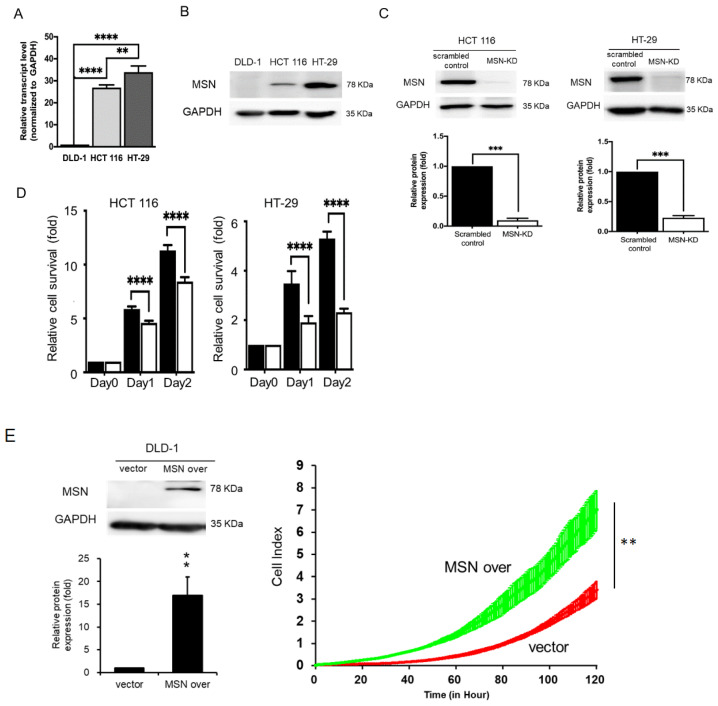
MSN promoted CRC cell proliferation. (**A**) The endogenous MSN expression level was analyzed through RT-qPCR. (**B**) Western blotting of MSN expression in CRC cell lines. (**C**) MSN was silenced in HCT 116 and HT-29 cells by using MSN-shRNA. The level of MSN was determined through RT-qPCR and Western blotting. (**D**) Silencing of MSN in HCT 116 and HT-29 cells reduced cell proliferation activity, as determined in the SRB assay. (**E**) MSN-overexpressing DLD-1 cells exhibited increased cell proliferation activity, as determined using the x’Cellgenes biosensor system. ** *p* < 0.01, *** *p* < 0.001, **** *p* < 0.0001.

MSN expression levels in three CRC cell lines (HT-29, HCT 116, and DLD-1) were determined by RT-qPCR and Western blotting to determine the possible regulation of MSN in these cell lines. MSN expression was found to be higher in HCT 116 and HT-29 cells than in DLD-1 cells (Figure 2A,B). We also performed a loss-of-function analysis in HCT 116 and HT-29 cells, as well as a gain-of-function analysis in DLD-1 cells. Western blotting was used to confirm MSN expression levels in stable cell lines. MSN expression was successfully inhibited by more than 85% in HCT 116 and HT-29 cells (Figure 2C), and MSN was overexpressed (MSN over) in DLD-1 cells (Figure 2E). To investigate the role of MSN in CRC cell proliferation, we silenced MSN (MSN-KD) in HCT 116 and HT-29 cells and measured their growth ability using an SRB assay. MSN silencing reduced HT-29 and HCT 116 cell proliferation (Figure 2D). When compared to vector control cells, MSN-overexpressing cells grew faster (Figure 2E). These findings suggest that MSN plays a role in cell growth.

### 2.3. Cell Cycle Arrest in the G2/M Phase Was Caused by MSN Knockdown

**Figure 3:**
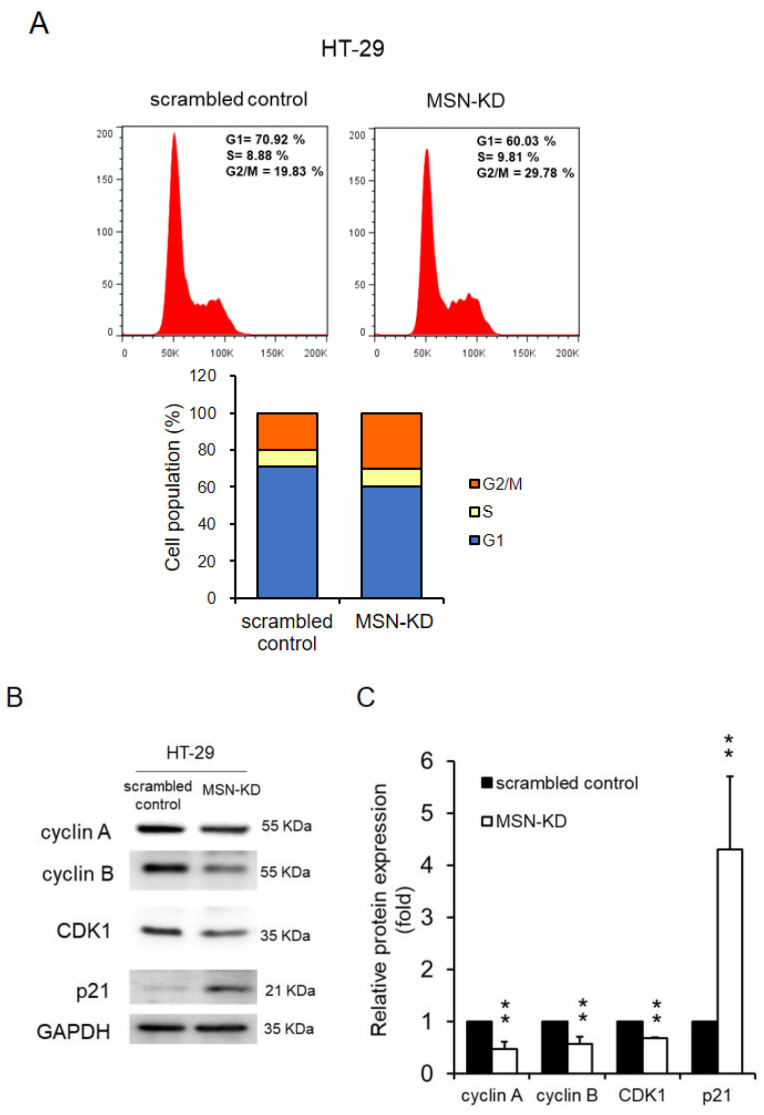
Knockdown of MSN arrests cell division at the G2M phase. (**A**) Silencing of MSN arrested the cell cycle at the G2M phase. (**B**) The expression levels of cyclin A, cyclin B, and cyclin D1 were determined through Western blotting. (**C**) Quantified cyclin A, cyclin B, and cyclin D1 levels were decreased in scrambled control cells, whereas the p27 level was increased in MSN-KD cells. ** *p* < 0.01.

The effects of MSN on cell cycle progression were investigated. MSN silencing caused cell cycle arrest in the G2/M phase in HT-29 cells, according to the findings (Figure 3A). Western blotting revealed that the expression levels of cyclin A, cyclin B, and cyclin D1 decreased, while p21 increased in MSN-KD cells (Figure 3B,C), indicating that MSN-KD cause G2/M phase cell cycle arrest.

### 2.4. MSN Knockdown Decreased Adhesion Activity

**Figure 4:**
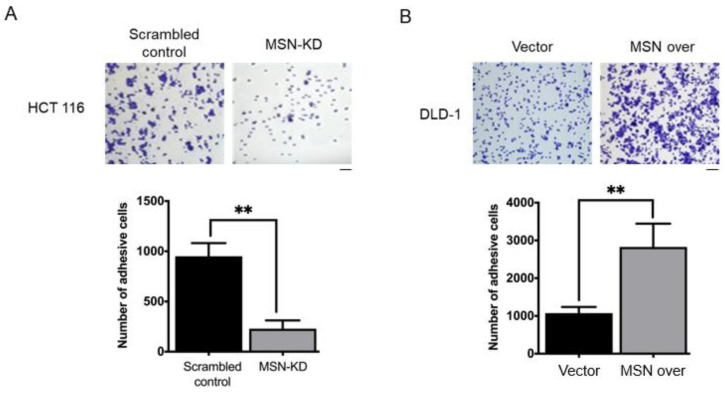
MSN increased cell adhesion activity in CRC cells. (**A**) Adhesion activity was decreased in MSN-KD HCT 116 cells. Quantified results are shown in the lower panel. (**B**) The adhesion ability was increased in MSN over DLD-1 cells. CRC cell adhesion activity was assessed 3 h after seeding cells on a 24-well plate. The attached cells were fixed and stained with crystal violet. Scale bar: 100 µm. Values are the means of three independent experiments. ** *p* < 0.01.

Since cell adhesion activity is linked to cancer progression and MSN connects the cell membrane and actin-based cytoskeleton, we investigated whether MSN influences cell adhesion activity. MSN silencing significantly decreased adhesion activity (Figure 4A), whereas MSN overexpression significantly increased adhesion activity (Figure 4B).

### 2.5. MSN Knockdown Decreased CRC Cell Invasion and Migration

**Figure 5:**
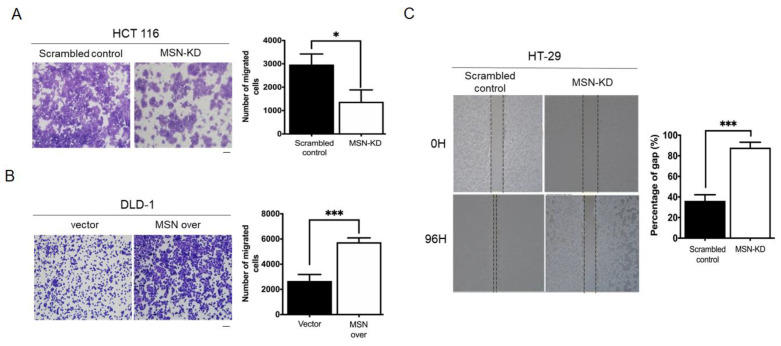

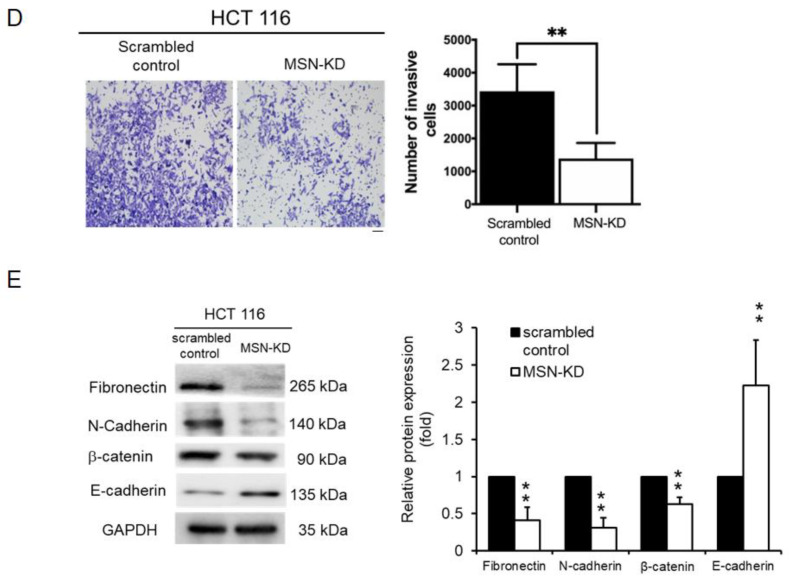
Knockdown of MSN suppressed the migration and invasive ability of CRC cells. (**A**) Migration ability was determined using the Transwell migration assay in scrambled control and MSN-KD HCT 116 cells. The quantified result indicated a decrease in silenced MSN HCT 116 cells. Scale bar: 100 µm (**B**) Overexpression of MSN increased the migratory ability of DLD-1 cells and quantified result of control and MSN over DLD-1 cells. Scale bar: 100 µm (**C**) The wound healing assay was performed to determine the wound-healing migration activity of scrambled control and MSN-KD cells in HT-29 cells. The quantified result indicated that the wound healing migration was reduced after silencing of MSN. (**D**) The invasive ability of MSN-KD cells in HCT 116 was determined by performing the invasion assay. The quantified result indicated that the invasive ability was reduced after silencing of MSN. Scale bar: 100 µm (**E**) EMT markers were checked through Western blotting. * *p* < 0.05, ** *p* < 0.01, *** *p* < 0.001.

We used migration and invasion assays to see if MSN increased the metastatic potential of CRC cells. MSN-KD cells showed decreased migration ability, which was opposite in MSN-overexpressing cells (Figure 5A,B). A wound healing assay was also performed to determine the migratory ability of scrambled control and MSN-KD HT-29 cells. As illustrated in Figure 5C, MSN silencing significantly reduced the wound healing ability. We also tested the invasion ability of scrambled control and MSN-KD cells. MSN-KD cells had a reduced ability to invade (Figure 5D). Changes in EMT biomarkers, as we know, contribute to cancer metastasis. We used Western blotting to investigate EMT biomarkers. MSN knockdown decreased mesenchymal markers such as fibronectin, N-cadherin, and vimentin while increasing epithelial markers such as E-cadherin (Figure 5E). MSN regulates CRC cell migratory ability via EMT biomarkers, according to our findings.

### 2.6. MSN Silencing Reduced RUNX2 Expression in CRC Cells

**Figure 6:**
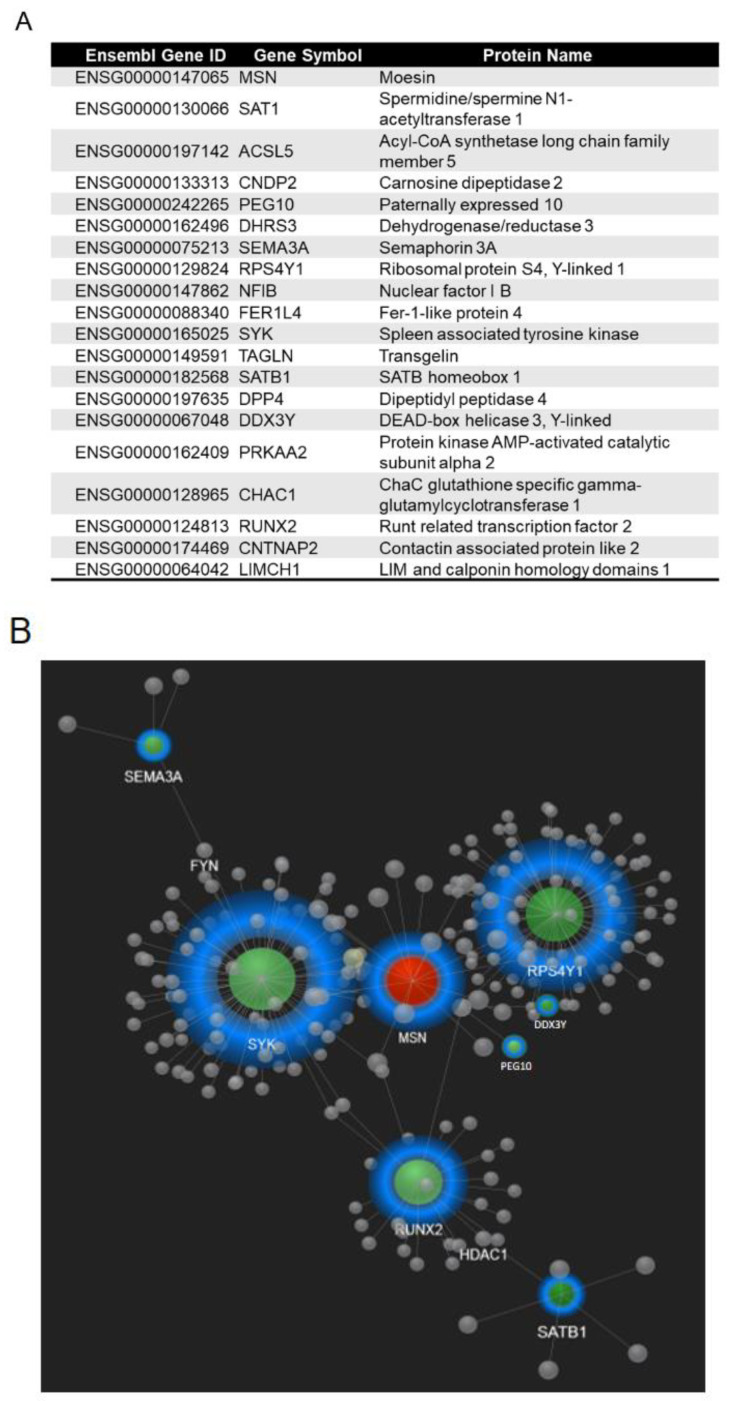
Differentially expressed genes and protein–protein interaction network. (**A**) Differentially expressed genes with log FC changes ≥ 2 (*p* ≤ 0.05) between HCT 116 control and MSN-KD cells by RNA-sequencing analysis. (**B**) Protein–protein interaction networks were created using the STRING interactome in the NetworkAnalyst 3.0 platform Red indicates upregulated genes, whereas green indicates downregulated genes.

We used RNA-seq to look at the identified candidate genes in order to determine how MSN regulates CRC progression. To identify significant differences in gene expression (*p* 0.05), differential gene expression (log-fold change 2) was examined. When we compared scrambled control and MSN-KD HCT 116 cells, we found 20 significant DEGs (Supplemental Table S1). SAT1, ACSL5, CNDP2, PEG10, DHRS3, SEMA3A, RPS4Y1, NFIB, FER1L4, SYK, TAGLN, SATB1, DPP4, DDX3Y, PRKAA2, CHAC1, RUNX2, CNTNAP2, and LIMCH1 expression levels were reduced when MSN was silenced (Figure 6A). PEG10, SEMA3A, RPS4Y1, SYK, SATB1, DDX3Y, and RUNX2 were found to be involved in protein–protein interactions in the MSN downstream signaling pathway according to STRING Interactome network analysis (Figure 6B). Furthermore, in the cBioPortal database, RUNX2 was the most significantly positively correlated with MSN expression in CRC patients among the seven key genes (Supplemental Figure S1).

### 2.7. MSN Controlled RUNX2 via the Wnt/β-catenin Signaling Pathway

**Figure 7:**
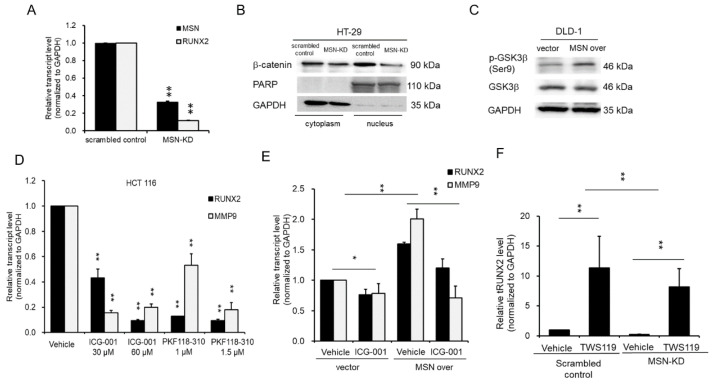
MSN upregulated RUNX2 expression in CRC cells. Expression levels of MSN and RUNX2 were determined through RT-qPCR. (**A**) Silencing of MSN reduced the expression of RUNX2 in HCT 116 cells. (**B**) The nuclear translocation of β-catenin was determined through Western blotting of cytoplasmic and nuclear extracts. PARP and GAPDH were used as loading controls for nuclear and cytoplasmic fractions, respectively. (**C**) Levels of Phospho-GSK3β-S9 and total GSK3β were determined in vector and MSN over DLD-1 cells through Western blotting. GAPDH is shown as a loading control. RUNX2 and MMP9 were evaluated through RT-qPCR in the presence of β-catenin inhibitors, ICG-001 and PKF118-310, in HCT 116 cells (**D**) and vector and MSN-overexpressing DLD-1 cells (**E**). RUNX2 expression was determined through RT-qPCR in scrambled control and MSN-KD HCT 116 cells after treatment with a GSK3β inhibitor, TWS 119 treatment (**F**). * *p* < 0.05, ** *p* < 0.01.

**Figure 8:**
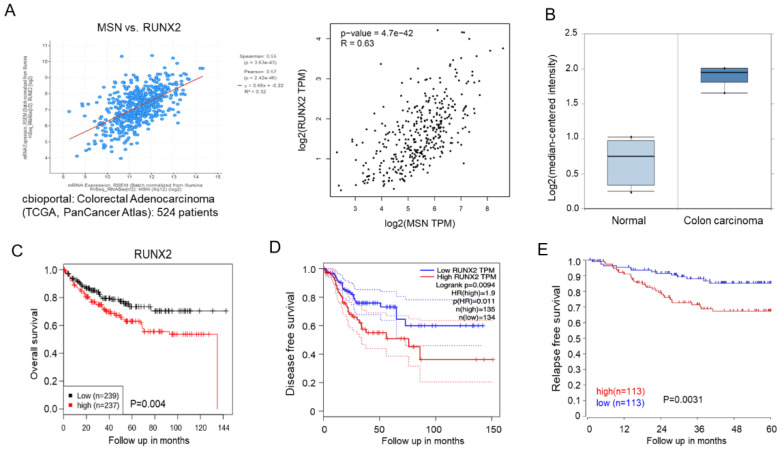
RUNX2 expression was positively correlated with the MSN level and poor prognosis in patients with CRC. (**A**) The correlation of RUNX2 and MSN expression level was analyzed using cBioportal and GEPIA databases. (**B**) RUNX2 was increased in tumor tissues in CRC by oncomine (*p* < 0.001). The prognostic value of RUNX2 in overall survival (**C**), disease-free survival (**D**), and relapse-free survival (**E**) in patients with CRC was analyzed using the GENT2 dataset (*p* = 0.04), GEPIA, and R2 dataset, respectively.

We used RT-qPCR to determine RUNX2 mRNA expression level in MSN-KD cells to validate the RNA-seq and bioinformatics results. RUNX2 levels in MSN-KD cells were lower than in scrambled control cells (Figure 7A). β-catenin promotes RUNX2 transcription [39]. We discovered a decrease in β-catenin expression in MSN-KD cells (Figure 5E). To investigate the MSN signal transduction pathway involved in RUNX2 regulation, we determined the subcellular localization of β-catenin as well as the expression of GSK-3beta (GSK3β), a key player in β-catenin degradation. MSN silencing lowers cytoplasmic and nuclear β-catenin levels. Internal controls included cytosolic GAPDH and nuclear PARP expression (Figure 7B). MSN-overexpressing cells had higher levels of phospho-GSK3 (Ser9), the inactive form of GSK3β, than vector control cells (Figure 7C). To test whether MSN regulates RUNX2 expression via β-catenin, we used the β-catenin inhibitor ICG-001 and the β-catenin/T-cell factor inhibitor PKF118-301 on cells. As illustrated in Figure 7D, the levels of RUNX2 and MMP9 were reduced in response to β-catenin inhibitor treatment. Furthermore, we treated vector control and MSN-overexpressing cells with β-catenin inhibitors (ICG-001) and discovered that ICG-001 treatment reduced RUNX2 levels in both vector control and MSN-overexpressing cells. MSN-overexpressing cells had a greater fold change in RUNX2 level than vector control cells. MMP-9, a transcriptional target of Wnt/β-catenin signaling, exhibited the same pattern as RUNX2 (Figure 7E). Furthermore, we wanted to see if MSN regulates RUNX2 expression via the GSK3β–β-catenin axis. TWS 119, a GSK3β inhibitor, was used to treat MSN-KD cells. As illustrated in Figure 7F, RUNX2 expression was significantly increased in both scrambled control and MSN-KD cells after TWS 119 treatment. The RNUX2 fold change was greater in scrambled control cells than in MSN-KD cells. Furthermore, in the cBioPortal and GEPIA databases, RUNX2 expression was found to be positively correlated with MSN expression in CRC patients (Figure 8A). RUNX2 expression levels in colon carcinoma tissues were higher than in normal tissues (Figure 8B). Higher RUNX2 expression levels were linked to poor overall, disease-free, and relapse-free survival in CRC patients (Figure 8C–E).

### 2.8. RUNX2 Silencing Reversed MSN’s Tumor-Promoting Effect in CRC Progression

**Figure 9:**
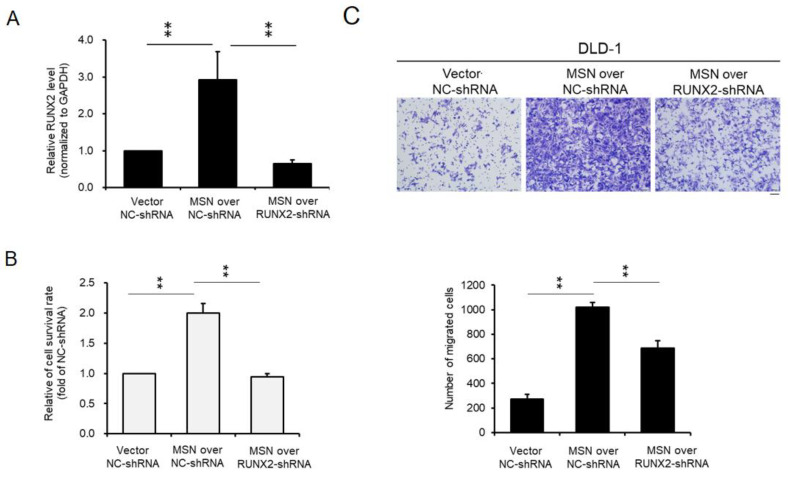
Silencing of RUNX2 reversed the gain-of-function role in MSN-overexpressing CRC cells. The RUNX2 levels of control, MSN-overexpressing–NC shRNA, and MSN-overexpressing–RUNX2 shRNA DLD-1 cells were detected through RT-qPCR (**A**). The proliferation of control-NC shRNA, MSN-overexpressing–NC shRNA, and MSN-overexpressing–RUNX2 shRNA DLD-1 cells was determined using the SRB assay (**B**). (**C**) The migratory ability of control–NC shRNA, MSN-overexpressing–NC shRNA, and MSN-overexpressing–RUNX2 shRNA DLD-1 cells was determined using the Transwell migration assay (upper panel), and the quantified results are shown (lower panel). All the experiments were repeated independently three times. Scale bar: 100 µm. ** *p* < 0.01.

To investigate MSN’s tumor-promoting effect on CRC via RUNX2, we silenced RUNX2 expression in MSN-overexpressing cells by transfecting shRNA against RUNX2 (RUNX2-shRNA). To generate control cells, control shRNA (NC-shRNA) was transfected into MSN-overexpressing cells. RT-qPCR was used to examine RUNX2 levels in NC-shRNA, MSN-overexpressing + NC-shRNA, and MSN-overexpressing + RUNX2-shRNA cells. MSN-overexpressing + RUNX2-shRNA cells had significantly lower RUNX2 expression than MSN-overexpressing + NC-shRNA cells (Figure 9A). Cell proliferation and migration were then examined in vector + NC-shRNA, MSN-overexpressing + NC-shRNA, and MSN-overexpressing + RUNX2-shRNA cells. RUNX2 silencing in MSN-overexpressing cells significantly reduced cell proliferation (Figure 9B). Furthermore, when MSN-overexpressing + RUNX2-shRNA cells were compared to MSN-overexpressing + NC-shRNA cells, migration activity was reduced (Figure 9C). Our findings suggest that MSN promotes CRC cell proliferation and migration via RUNX2.

## 3. Discussion

MSN has been linked to tumor growth, metastasis, invasion, and drug resistance in various types of cancer [15,19]. This study investigated MSN’s role in CRC. First, MSN was found to be highly expressed in CRC tissues, and it was linked to poor overall, disease-free, and relapse-free survival (Figure 1). Furthermore, silencing MSN decreased cell proliferation, adhesion, migration, and invasion. These findings suggest that MSN plays a role in the progression of CRC (Figure 2, Figure 3, Figure 4 and Figure 5). A previous study found that the decrease of p38 and ERM proteins inhibited cell proliferation in prostate cancer [40]. The same phenomenon was observed in rhabdomyosarcoma and breast cancer where silencing of ERM proteins suppressed tumor growth [41,42], and high MSN expression was associated with increased proliferation, migration, and invasion of glioblastoma cells [43]. Furthermore, our findings demonstrated that silencing of MSN suppressed CRC cell proliferation through cell cycle arrest (Figure 3). Early recurrence in advanced-stage CRC was correlated with low expression of ER and high expression of cyclin D1 [44]. Cell cycle arrest in the S/G2 phase increases cancer response to different therapies [45,46,47]. Because CRC is mostly resistant to current therapies [48,49], the identification of MSN as a potential treatment target can offer choice. MSN may be a good candidate for the prevention of early disease recurrence and distant metastases because it is involved in cell migration and invasion as well as mesenchymal marker regulation.

Using RNA-seq, this study determined the role of MSN in CRC; the levels of PEG10, RUNX2, and SEMA3A were highly correlated with the MSN level. These genes were linked to poor overall survival (OS) and disease-free survival, but RUNX2 was the only one linked to poor OS in patient tissues. RUNX2 has been shown to be regulated by β-catenin. Although this is the first study to show a link between MSN, RUNX2, and β-catenin, it supports previous findings that RUNX2 is involved in CRC progression and is associated with poor survival [33,34]. RUNX2 loss caused resistance to MEK inhibitors via RTKs in CRC with KRAS alterations [38].

In glioblastoma, increased MSN expression increased the expression of β-catenin, CD44, ICAM-1, and MMP-2.28. Increased MSN expression activates the Wnt/β-catenin pathway and results in aggressive orthotopic glioblastoma development in mice [28]. The Wnt pathway is important in many cancers, including head and neck squamous cell carcinoma [50], ovarian cancer [51], hepatocellular carcinoma [52], gastric adenocarcinoma, [53] prostate cancer [54], and CRC [55]. Together with the lymphoid enhancer factor/T-cell factor, β-catenin translocate to the nucleus and induces the transactivation of target genes [56]. These target genes frequently include cell adhesion molecules (CAMs) that induce the generation of CSCs [57].

**Figure 10:**
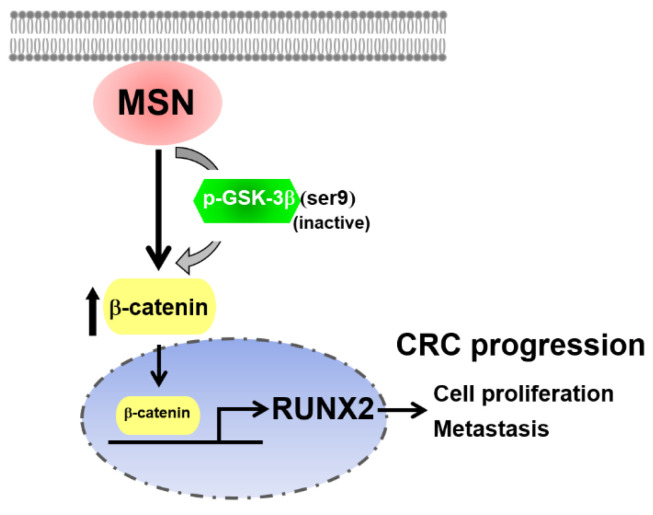
The scheme plot illustrates the role and signaling pathway of MSN in regulating CRC progression.

In CRC, an activated Wnt/β-catenin pathway caused resistance to chemotherapy, while the silencing of β-catenin sensitized CRC cells to chemotherapy [1,58]. ERM-binding phospho-protein 50 overexpression inhibited pancreatic cell proliferation and invasion via the Wnt/β-catenin and E-cadherin pathways [59]. The formation of invadopodia was increased by a higher MSN level, which activated the β-catenin–MMP9 axis in hepatocellular carcinoma [21]. Thus, we examined the β-catenin level in MSN-KD cells and found a decreased level of β-catenin in both the cytoplasm and nuclear parts (Figure 7B). A β-catenin inhibitor was used to treat control and MSN-overexpressing cells to determine if the RUNX2 level was induced by β-catenin. RUNX2 and MMP-9 levels were reduced after treatment with a β-catenin inhibitor (Figure 7D). JMJD1A activated Wnt/β-catenin signaling in CRC [60]. The overexpression of Rab1B and MMP-9 in CRC tissues was associated with metastasis, advanced tumor stage, and poor OS [61]. MMP-9, MMP-2, and β-catenin levels were elevated in CRC [62]. MMP-2, MMP-7, and MMP-9 promoter polymorphism was involved in CRC in the Kashmiri population [63]. We used β-catenin inhibitors to confirm that MSN is involved in the regulation of RUNX2 (Figure 7). RUNX2 expression was reduced after MSN-overexpressing cells were treated with β-catenin inhibitors (Figure 7E). Furthermore, we discovered a linear correlation between RUNX2 and MSN in CRC specimens (Figure 8). Finally, we observed that the knockdown of RUNX2 in MSN-overexpressing cells resulted in decreased cell survival and migration abilities, indicating the significant role of RUNX2 as a downstream effector of MSN (Figure 9). Our findings suggest that MSN facilitates the progression of colorectal cancer by activating the β-catenin–RUNX2 axis, highlighting the potential of MSN as a promising therapeutic target for treating CRC (Figure 10).

## 4. Materials and Methods

### 4.1. Chemicals, Reagents, and Cell Culture

Human colon adenocarcinoma cell lines including DLD1 (CCL-221), HT-29 (HTB-38), and HCT116 (CCL-247) were obtained from American Type Culture Collection (ATCC, Rockville, MD, USA). All cells were cultured in RPMI 1640 medium supplemented with 10% fetal bovine serum (FBS) (SAFC Biosciences, Lenexa, KS, USA) and 1% penicillin/streptomycin containing 100 IU/mL of penicillin and 100 μg/mL of streptomycin at 37 °C in 5% CO_2_ in a humidified incubator.

### 4.2. Transfection and Generation of Stable Colonies

To knock down MSN expression, short hairpin RNA (shRNA; TRCN0000062411 and TRCN0000062412) targeting human MSN NM_002444 was purchased from the National RNAi Core Facility at Academia Sinica in Taiwan. MSN-shRNA and nontarget shRNA were transfected into HCT 116 and HT-29 cells, and stably transfected cells were selected using puromycin for 2 weeks. The MSN level was determined through Western blotting and quantitative reverse transcription polymerase chain reaction (RT-qPCR). To overexpress MSN, the pCMV6-Entry-MSN (CAT No.: RC205674, OriGene Technologies, Inc., Rockville, MD, USA) was transfected into DLD-1 cells through electroporation. Subsequently, G418 was added to obtain stably transfected DLD-1 cells. The cells were used for subsequent experiments after confirming MSN overexpression through RT-qPCR and Western blotting.

### 4.3. RT-qPCR

Total RNA was extracted by using RNAzol in accordance with the manufacturer’s protocol (Molecular Research Center, Inc., Cincinnati, OH, USA). Reverse transcription of total RNA was performed using the Reverse Transcription ABI kit (Applied Bio Systems, Foster City, CA, USA). RT-qPCR of MSN, RUNX2, MMP-2, MMP-9, and GAPDH (internal control) was performed using 200 ng of complementary DNA, 0.5 µM of forward and reverse primers, and 2× SYBR Green Master Mix (Bio-Rad Laboratories, Hercules, CA, USA) in a final volume of 10 μL. The following primers were used: MSN, AATGCGCTGCTTGGTGTTG (forward), and TGGGCCGAGACAAATACAAGAC (reverse); RUNX2, TCGAATGGCAGCACGCTAT (forward), and TGGCTTCCATCAGCGTCAA (reverse); MMP-2, CCGCAGTGACGGAAAGATGT (forward), and GCCCCACTTGCGGTCAT (reverse); MMP-9, CCCTGGAGACCTGAGAACCA (forward), and CCACCCGAGTGTAACCATAGC (reverse); and GAPDH, CCTGTACGCCAACACAGTGC (forward), and ATACTCCTGCTTGCTGATCC (reverse). RT-qPCR was performed in triplicate for each sample.

### 4.4. Nuclear/Cytosol Extraction

The nuclear/cytosol fractionation kit from BioVision (Milpitas Boulevard, Milpitas, CA, USA) was used to separate the nuclear and cytoplasmic extracts of scrambled control and silenced MSN (MSN-KD) cells. According to the manufacturer’s instructions, 2 × 10^6^ cells were collected. Then, cytosol extraction buffer-B and nuclear extraction buffer mix were used to obtain cytoplasmic and nuclear extracts. Western blotting was performed to determine the protein levels of β-catenin, GAPDH, and PARP by using anti-β-catenin, GAPDH, and PARP antibodies.

### 4.5. Western Blotting

Protein lysates were prepared by suspending the cells in lysis buffer (Sigma-C2978) containing protease inhibitors (Boehringer Mannheim Indianapolis, Indianapolis, IN, USA). The cell lysates were centrifuged at 13,000 rpm for 15 min, and the supernatant was collected. The protein level (µg/mL) was measured using a protein assay kit (Bio-Rad Laboratories); the absorbance of the sample was determined at 595 nm by using a Bio-Rad Model 680 microplate reader. Aliquots of cell lysates containing 20 µg of total protein were subjected to sodium dodecyl sulfate–polyacrylamide gel electrophoresis on 10% acrylamide gel and were transferred to a polyvinylidene fluoride membrane (Pall Corp., Port Washington, NY, USA). The membranes were probed using the following primary antibodies at 4 °C overnight: GAPDH (sc-32233, Santa Cruz Biotechnology, Dallas, TX, USA), MSN (ab52490, Abcam PLC, Cambridge, UK), cyclin A (iR115, iReal Biotechnology Co., Hsinchu City, Taiwan), cyclin B (GTX10091), cyclin D (iR117-294, iReal Biotechnology Co., Hsinchu City, Taiwan), p21 (MCA2325, Bio-Rad Laboratories, Hercules, CA, USA), fibronectin (sc9068, Santa Cruz Biotechnology, Santa Cruz, CA, USA), N-cadherin (13116, Cell Signaling Technology, Danvers, MA, USA), vimentin (iR45-137, iReal Biotechnology Co., Hsinchu City, Taiwan), E-cadherin (3195, Cell Signaling Technology, Danvers, MA, USA), β-catenin (sc-7963, Santa Cruz Biotechnology, Santa Cruz, CA, USA), and PARP (sc-25780, Santa Cruz Biotechnology, Santa Cruz, CA, USA). Primary antibody reactivity was detected using horseradish-peroxidase-conjugated donkey anti-mouse and anti-goat secondary antibodies (Santa Cruz Biotechnology, Santa Cruz, CA, USA) and visualized using the SuperSignal West Pico Chemiluminescent Substrate (Pierce, Waltham, MA, USA) and Versa Doc Imaging system.

### 4.6. Cell Proliferation Analysis

The cells (5 × 10^3^) were seeded into 96-well plates and incubated at 37 °C in a 5% CO_2_-humidified incubator for 24, 48, and 72 h. Then, the cells were fixed with 10% trichloroacetic acid overnight at 4 °C and stained with 0.4% *w*/*v* protein-bound sulforhodamine B (SRB) for 30 min at room temperature. Next, the stained cells were washed twice with 1% acetic acid. After air drying overnight, the protein-bound dye was dissolved in 10 mM Tris base solution, and absorbance was measured at 515 nm by using a microplate reader (Bio-Rad Laboratories). The xCELLigence Real-Time Cell Analysis (RTCA) Dual-Purpose instrument (ACEA Biosciences, Inc., San Diego, CA, USA) was employed to analyze cell proliferation and migration as previously described [64]. The cell growth rate was determined using an E-plate 16 (ACEA Biosciences, Inc.). Cells were monitored once every 30 s for 4 h. After being seeded on an E-plate in FCS-containing medium at a density of 5000 cells per well, they were monitored every 30 min. The data were analyzed using RTCA software 1.2 (supplied with the instrument).

### 4.7. Colony Formation Analysis

We seeded 1 × 10^3^ cells (HCT 116 scrambled control, HCT 116 MSN-KD, DLD-1 control, and MSN-overexpressing (MSN over) DLD-1 cells) into 6-well plates individually and incubated them in a 5% CO_2_ incubator at 37 °C without changing the culture medium. After 10 days, the cells were fixed with 0.4% formaldehyde and stained with 0.1% crystal violet. The number of colonies was counted using ImageJ (version 1.53) or a hand counter.

### 4.8. Adhesion Assay

We seeded 16 × 10^4^ cells (scrambled control, MSN-KD HCT 116, control, and MSN over DLD-1 cells) into 24-well plates individually. After 3 h, the cells were gently washed with phosphate-buffered saline (PBS) and stained with 0.1% crystal violet for 1 h. Images were obtained at 10× magnification by using an Olympus IX microscope, and the number of cells was counted using ImageJ or a hand counter.

### 4.9. Cell Cycle Analysis

We seeded 6 × 10^5^ cells (scrambled control and MSN-KD HCT 116 cells) into 6 cm dishes and incubated them at 37 °C in a 5% CO_2_ incubator for 48 h. The cells were collected and washed twice with PBS. Then, the cells were fixed with 70% ethanol and washed twice with PBS. Subsequently, 0.1% Triton X and RNase were added into propidium iodide (PI) before use. The cells were stained with PI in the dark. The cells were analyzed using a flow cytometer (BD Biosciences, San Jose, CA, USA), and data were evaluated using FLOWJO software (version 7.6.2).

### 4.10. Transwell Migration Assay/Invasion Assay

In vitro cell migration and invasion assays were performed using a BD Falcon cell culture insert and BD BioCoat Matrigel invasion chambers precoated with BD Matrigel matrix, respectively (BD Biosciences). Aliquots of 1 × 10^5^ cells suspended in 500 μL of serum-free RPMI medium were seeded into the upper compartment of each chamber, and the lower compartments were filled with 1 mL of RPMI medium containing 10% fetal bovine serum and 1% penicillin and streptomycin. After incubation for 48 h at 37 °C in a 5% CO_2_ incubator, each well and chamber was washed once with 1 mL of 1× PBS. The cells were fixed in less than 1 mL of methyl alcohol solution for a few seconds. Nonmigrated cells were mechanically removed from the upper surface of the membrane. The cells on the reverse side were stained with 0.1% crystal violet. After the plate was incubated at room temperature for 8 h, crystal violet was removed, and the number of stained cells was counted using a microscope (Olympus IX) at 10-fold magnification. The number of migrated cells was counted using a handheld cell counter.

### 4.11. Wound Healing

The migration of scrambled control/HT-29 MSN-KD cells was determined in real time by using the wound-healing assay (Luma System). We prepared a suspension of 5 × 10^5^ cells/mL, and 70 μL of this suspension was seeded into Ibidi culture inserts (Ibidi GmbH, Münich, Germany) placed at the bottom of a 35 mm dish and then incubated at 37 °C overnight. After overnight incubation, the culture inserts were removed, and the 35 mm dish was filled with 3 mL of fresh medium. The dish was placed into a stage-top incubator on an inverted microscope, and the cell-free gap was observed using a 20× objective lens.

### 4.12. NetworkAnalyst 3.0: A Visual Analytics Platform for Comprehensive Gene Expression Profiling and Meta-Analysis

RNA sequencing (RNA-seq) was performed to identify differentially expressed genes (DEGs) between scrambled control and MSN-KD cells. Log FC changes ≥ 2 and *p* ≤ 0.05 were set as the cutoffs. We identified 20 significant DEGs when comparing scrambled control cells with MSN-KD HCT 116 cells. Volcano plots were created to determine changes in gene expression profiles. The NetworkAnalyst 3.0 platform was used to analyze the RNA-seq results [65]. The STRING interactome on this platform was used to determine protein–protein interaction networks [66]. A value of 0.9 (high confidence) was considered as the cutoff.

### 4.13. Overall Survival, Disease-Free Survival, and Relapse-Free Survival

DEGs involved in the pathways were clinically validated using GENT2 [67] (GSE database) and the R2 Genomics Analysis and Visualization Platform (http://r2.amc.nl). Average expression levels of all target genes in normal versus tumor tissues were determined using ONCOMINE (https://www.oncomine.org/resource/login.html, accessed on 10 November 2021). The association of the expression levels of these genes with overall survival (OS) in months was independently evaluated using GENT2 (http://gent2.appex.kr/gent2/, accessed on 11 August 2022) and oncogenomics (https://hgserver1.amc.nl/cgi-bin/r2/main.cgi, accessed on 11 August 2022) tools by plotting Kaplan–Meier survival curves. Hazard ratios (HRs) with 95% confidence intervals (CIs) were calculated. A log-rank *p* value of <0.05 was considered statistically significant.

### 4.14. cBioPortal Database

cBioPortal is an open-access resource that can be used to search for multidimensional cancer genomics datasets (http://www.cbioportal.org/, accessed on 12 July 2022) [68,69]. cBioPortal online analysis was used to predict the genes coexpressed with MSN.

### 4.15. Statistical Analysis

Data are presented as the mean ± standard deviation of at least three independent experiments. Significant differences between groups were analyzed using Student’s *t* test (two tailed). A *p* value of <0.05 was considered statistically significant.

## 5. Conclusions

This study revealed the novel role of MSN in CRC. We discovered that MSN regulates CRC progression by regulating the β-catenin/RUNX2 pathway. Our analysis on publicly available clinical datasets further confirmed the association of high MSN as well as high RUNX2 expression with poor patient survival. We found that MSN regulated RUNX2 though β-catenin. Furthermore, we confirmed that MSN-induced CRC progression can be abrogated by inhibiting either β-catenin or RUNX2. Moreover, several studies have already been conducted to determine the effect of the β-catenin inhibitor as a chemotherapeutic choice. Thus, together, the findings of our study emphasize the targeting of MSN in CRC treatment and suggest that the development of therapeutics for colorectal cancer patients with high MSN expression could enhance treatment efficacy.

## Data Availability

The dataset supporting the conclusions of this article are included within the article.

## Supplementary Materials

The following supporting information can be downloaded at: https://www.mdpi.com/article/10.3390/ijms241310951/s1.

## Abbreviations

Colorectal cancer: CRC; moesin: MSN; ezrin–radixin–moesin: ERM; Runt-related transcription factor: RUNX; silenced MSN: MSN-KD; cell adhesion molecules: CAMs.
