# Supplementary material for: Identification of Moesin (MSN) as a Potential Therapeutic Target for Colorectal Cancer via the β-Catenin-RUNX2 Axis
Source: Int J Mol Sci. 2023 Jun 30;24(13):10951. doi: 10.3390/ijms241310951 (PMC10341927; doi:10.3390/ijms241310951)
Supplement: Supplementary file 1 [file ijms-24-10951-s001.zip › ijms-2385201-supplementary.pdf]

Supplemental Table S1. Differentially expressed genes.

No	Symbol	Log2fold	padj
1	SAT1	−2.269	3.1E-135
2	MSN	−2.828	6.85E-44
3	ACSL5	−2.542	8.59E-39
4	CNDP2	−2.941	2.07E-17
5	PEG10	−2.903	1.25E-14
6	DHRS3	2.300	1.95E-11
7	SEMA3A	−3.984	9.86E-06
8	RPS4Y1	−5.613	1.94E-05
9	NFIB	−6.721	3.87E-05
10	FER1L4	−2.457	0.000556
11	SYK	−3.699	0.001409
12	TAGLN	2.685	0.001856
13	SATB1	−6.533	0.004604
14	DPP4	−2.605	0.007916
15	DDX3Y	−4.681	0.008699
16	PRKAA2	−4.541	0.015905
17	CHAC1	2.155	0.016749
18	RUNX2	−3.061	0.0239
19	CNTNAP2	−3.440	0.026913
20	LIMCH1	−2.127	0.030347

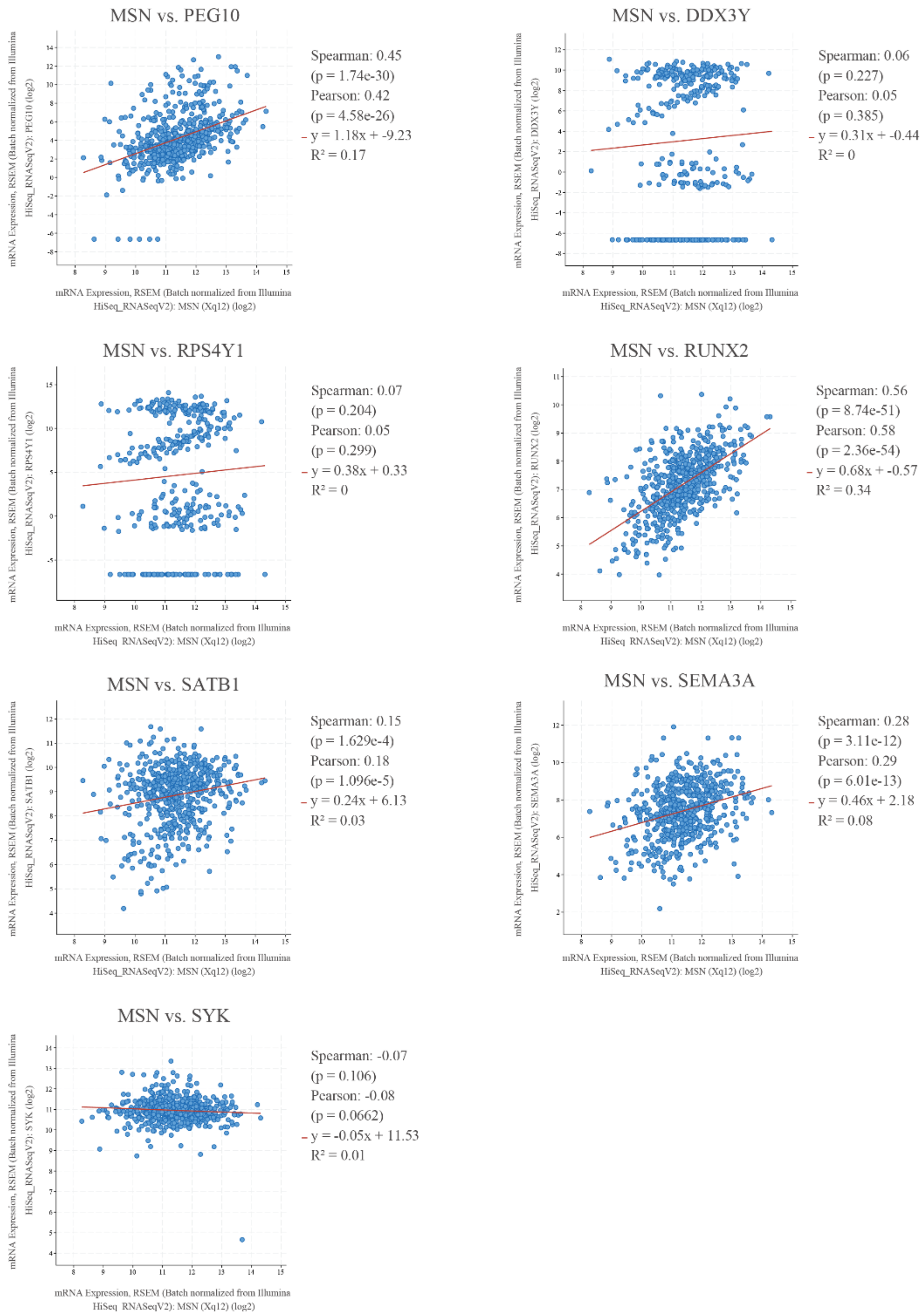

Supplemental Figure S1. RUNX2 was the most significantly positively correlated with MSN expression in CRC patients. The expression correlation of the seven key genes (PEG10, SEMA3A, RPS4Y1, SYK, SATB1, DDX3Y, and RUNX2) with MSN was analyzed by the cBioPortal database.
